# Hysteresis-assisted shape morphing for soft continuum robots

**DOI:** 10.1126/sciadv.adx3024

**Published:** 2025-10-15

**Authors:** Zheyuan Bi, Tianchen Ji, Sanja Dogramadzi, Soo Jay Louis Phee, Jiajun Liu, Wenjie Lai, Diyang Wu, Bing Zhang, Lin Cao

**Affiliations:** ^1^ School of Electrical and Electronic Engineering, The University of Sheffield, Sheffield, UK.; ^2^ School of Mechanical and Aerospace Engineering, Nanyang Technological University, Singapore, Singapore.; ^3^ Singapore-HUJ Alliance for Research and Enterprise (SHARE), Campus for Research Excellence and Technological Enterprise (CREATE), Singapore, Singapore.; ^4^ School of Mechatronic Engineering and Automation, Shanghai University, Shanghai, China.

## Abstract

Conventional robots require more actuators for greater dexterity, increasing design and control complexity. We introduce a hysteresis-assisted shape-morphing (HasMorph) paradigm for soft continuum robots and integrate with tip-everting soft-growing robots. Unlike conventional approaches that avoid hysteresis, HasMorph intentionally exploits friction-induced shape hysteresis, the difference in robot shape during loading and unloading, to enable numerous reversible shape changes using only two sequentially controlled actuators via an inverted zigzag tendon-sheath mechanism. Integrated with tip growth, the robot achieves complex, reversible morphologies and frictionless follow-the-leader navigation. Mechanics and kinematics models reveal how friction and input sequences govern shape evolution and expand the robot’s configuration space. Experiments validate dexterous morphing and robust navigation in unstructured environments. HasMorph enables direct, fine-grained actuation of hyperredundant soft bodies with minimal actuation, offering a scalable approach to soft robot design. This paradigm opens avenues for minimally invasive surgical tools and adaptive inspection systems in complex, confined spaces.

## Teaser

HasMorph leverages shape hysteresis in soft robots to enable billions of controlled shapes by alternating just two actuators.

## INTRODUCTION

Accessing and working in confined spaces inaccessible to humans has remained a symbolic challenge in robotics (*1*). Overcoming this challenge can enhance the feasibility, safety, efficiency, and cost-effectiveness of tasks in various sectors, such as healthcare for minimally invasive surgery (*2*–*4*), the water/oil/gas industries for underground pipeline maintenance (*5*, *6*), and the aerospace sector for maintenance of aircraft wings and engines (*7*, *8*).

Snake-like continuum robots, known for their shape adaptability, are promising for this challenge (*9*, *10*). However, continuum robots are mostly designed with the “more actuators for more dexterity” actuation paradigm, resulting in bulky designs, high costs, and control difficulties or a lack of dexterity (*11*–*14*). Alternative strategies like underactuated robots and single-input multimodal robots have been explored. Underactuated robots leverage system dynamics, passive elements, and environmental interactions to achieve more dexterity with fewer actuators (*15*, *16*), but their capabilities are limited by the requirements on dynamics or external interactions. Single-input multimodal robots use a single actuation input to drive multiple motion modes by using multistable structures (*17*–*19*), but these multistable structures are complex in design, fabrication, and integration and are still limited in achievable motion modes. As such, although a continuum robot has many passive degrees of freedom (DOFs), but how to actively actuate these DOFs effectively in a compact profile remains a challenge.

The same challenge is even more critical for the emerging tip-everting soft-growing robots (SGRs) that navigate in confined spaces through tip growth rather than conventional sliding insertion (*20*). An SGR lengthens its tip by everting out preinverted thin-film tubing under pneumatic or fluidic pressure. This tip-growth process features frictionless movement because there is no relative sliding between the robot and the environment, allowing SGRs to access challenging environments, such as tortuous pathways in medical diagnostics and pipeline inspection (*21*). However, steering the tip and reconfiguring the shape of the robot body are challenging because of the ever-growing robot body. As such, many SGR designs can only produce a single bend (*22*–*24*), limiting its dexterity and capabilities. Some approaches achieve complex multibend shapes but require many actuators (e.g., valves and tubes) and bulky designs (*25*), or the achievable shape is either irreversible (one-off) (*20*, *26*, *27*) or prescribed (*28*–*31*). A compact actuation mechanism that facilitates reversible multibend shapes and tip growth is needed.

Here, we introduce a hysteresis-assisted shape-morphing (HasMorph) actuation paradigm. HasMorph exploits shape hysteresis to achieve reversible, continuous, and multibend shape morphing of continuum robots using only a few actuators. Hysteresis refers to the phenomenon where a system’s output depends not only on its current input but also on its past states (*32*, *33*). In this context, shape hysteresis means that the robot’s shapes during the loading and unloading phases are different; alternating the loading and unloading sequences (and magnitudes) of a few actuators would generate numerous complex shapes that would, otherwise, require a large number of actuators for the conventional more actuators for more dexterity paradigm. In this work, HasMorph was achieved on an inflated continuum robot through an inverted zigzag tendon-sheath mechanism (TSM) that was routed in an inverted zigzag pattern from the tip to the base of the robot. This inverted zigzag TSM leads to shape hysteresis, e.g., different shapes at the tendon pull (loading) and release (unloading) phases, enabling numerous complex planar shapes by varying the actuation sequences and magnitudes of only two actuators. Furthermore, this compact inverted zigzag TSM was then used to reversibly steer the tip and reconfigure the body shape of an SGR during tip growth, enabling them to navigate tortuous turns and avoid obstacles while frictionlessly maneuvering in confined spaces.

The main contributions of this work are as follows: (i) The HasMorph concept: We leverage shape hysteresis to achieve multibend shape morphing using only a few actuators. It notably increases the configuration space and dexterity with a compact, low-cost design; in contrast, the conventional “more actuation for more dexterity” paradigm of robotics requires proportionally more actuators for more dexterity, which complicates design and limits dexterity. In addition, traditionally, hysteresis has been viewed as a drawback in robotics that causes actuation errors and control challenges (*34*, *35*). While prior studies focused on eliminating hysteresis effects (*36*, *37*), HasMorph leverages friction-induced hysteresis for shape morphing, providing a new, positive perspective to hysteresis. (ii) Inverted zigzag TSM: This mechanism leads to independent, local bending of each section of the robot by alternating the actuation sequences of its actuators. It can generate $3^{n}$ distinct configurations with only two tendons (*n* is the number of sections and theoretically has no limit); in contrast, with other tendon routings [straight or helical; (*38*, *39*)], the final configuration is prescribed or two tendons can drive only a single section with three configurations. (iii) Theoretical contributions: A mechanics model and a kinematics model were developed and verified to reveal the shape-morphing principle. (iv) Application demonstrations: We validate the HasMorph system through a series of navigation tasks in confined spaces, showcasing its potential for search and rescue, medical navigation, and industrial inspection.

## RESULTS

### Concepts and design principles

**Fig. 1.**
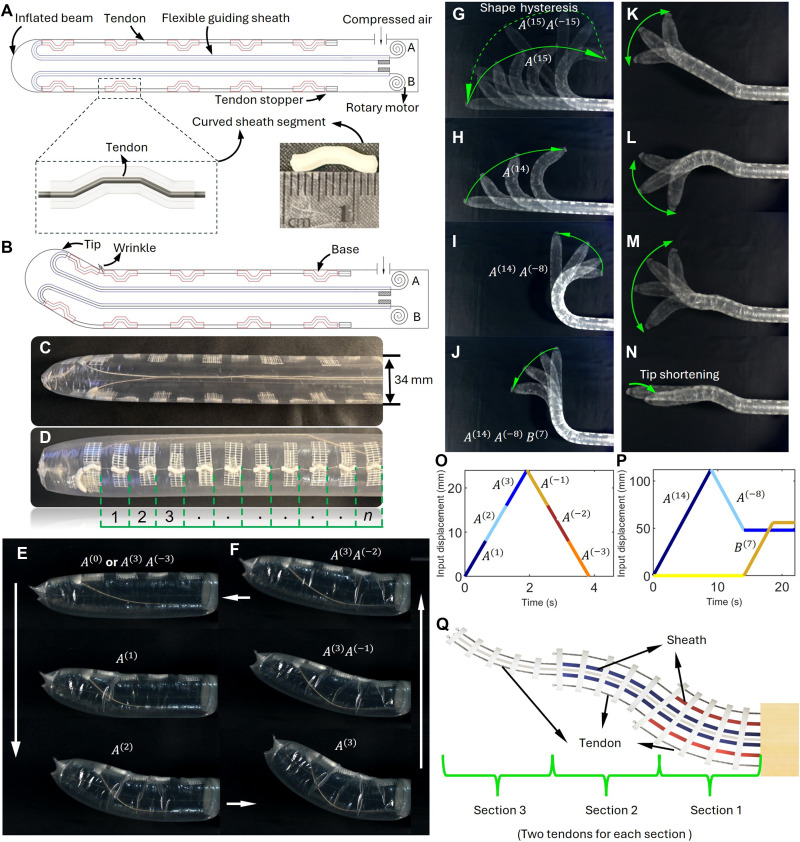
Working principle and prototype. (**A**) An inflated continuum beam driven by inverted zigzag TSMs features curved sheath segments attached serially along its inner wall with intervals. Each tendon is routed from the motor to the tip via the guiding sheath and then inverts to the base via the curved sheath segments. (**B**) The motor pulls the tendon, causing the inflated beam to sequentially bend from the tip to the base, section by section. (**C** and **D**) The robot prototype. (**E**) Sequential bending from the tip to the base, with gaps closing one by one (see movie S1). (**F**) Sequential unbending from the tip to the base, with gaps opening one by one. (**G**) Distinct paths during the loading and unloading phases, leading to shape hysteresis. Input commands for motion are defined as $A^{\left(n\right)}$ and $B^{\left(n\right)}$ , where positive n means closing n segments from the tip (tendon pull) and negative n means opening them (tendon release). (**H** to **J**) The robot transforms into an S-shape through the sequential actuation of the two motors. (**K** to **M**) Cases where the robot can be steered at the tip with the remaining body unaffected. (**N**) Pulling both tendons shortens the robot from the tip, useful for posture adjustment. Refer to movie S2 for actions shown in (G) to (N). (**O**) Input displacements corresponding to the cases in (E) and (F). (**P**) Input displacements corresponding to the case in (I) and (J) (simulation). (**Q**) A conventional three-section continuum robot driven by six tendons, with each section being independently actuated by two tendons. More dexterity would require more actuators, increasing system complexity.

Figure 1 presents the shape-morphing principle of the robot. As shown in Fig. 1A, an inflated beam is driven by two Inverted Zigzag TSMs with two crucial routing features: (i) each tendon goes through a flexible coiled guiding sheath from the base to the tip; and (ii) then returns to the base via a series of short, curved sheath segments attached to the inner wall of the inflated beam, forming an inverted zigzag tendon routing. The guiding sheath is a flexible and incompressible coil that enables all tendon displacement at the motor side to transmit to the tip of the robot. Pulling a tendon would let the inflated beam wrinkle (local buckling) and bend at the gaps between the curved sheath segments (Fig. 1B). The inset in Fig. 1A details the three-dimensionally (3D) printed curved sheath segment [polylactic acid (PLA)], which is attached to the inner wall of the inflated beam using strong filament tapes (see the prototype in Fig. 1C). Note that attaching the curved sheath segments to the inner wall of the inflated beam can make the outer surface of the robot smooth. The curved sheath segments divide the beam into n bending sections from the tip to the base (Fig. 1D) and induce friction on the tendon because of the curved feature, lowering the tendon tension from the tip to the base of the inflated beam (Fig. 1C). When pulling one tendon, bending starts at the far tip and propagates toward the base, section by section (Fig. 1E, “loading”); similarly, when releasing the tendon (Fig. 1F, “unloading”), the unbending starts at the far tip and propagates toward the base. This tip-to-base sequential motion leads to shape hysteresis: The shapes (and tip trajectories) of the robot during the loading phase (tendon pull) and unloading phase (tendon release) are different (Fig. 1G). Relying on this hysteresis mechanism, changing the pull/release sequences and magnitudes of the two tendons would generate numerous complex shapes with multiple bends (Fig. 1, H to J). Essentially, by alternating the actuation sequence and magnitudes of the two tendons, we are able to individually control the bending of each curved sheath segment.

The following conventions are used to denote actuation sequences: $A^{\left(n\right)}$ represents the input pull displacement of the tendon A to bend sections 1 to n , and $A^{\left(-n\right)}$ is the input release displacement to unbend sections 1 to n . For instance, $A^{\left(2\right)}$ is to pull the tendon A until section 2 bends. Because of the sequential motion, both section 1 and section 2 are bent due to the input $A^{\left(2\right)}$ [as demonstrated in Fig. 1 (E and F)]. Take the robot prototype as an example, tendon A is firstly pulled to bend the first 14 sections from the tip, as shown in Fig. 1H. Here, we denote the actuation as $A^{\left(14\right)}$ . Subsequently, in Fig. 1I, tendon A is released to unbend the first 8 of the 14 bent sections, indicated as $A^{\left(-8\right)}$ . As such, sections 9 to 14 are bent, while the other sections are not. A similar operation can be performed on tendon B to achieve the same effect, as shown in Fig. 1J, which corresponds to the input $B^{\left(7\right)}$ . Based on similar series of operations, each section i(i=1,…,n) can be independently controlled with three distinct configurations: positive/negative bends and straight. Thus, this mechanism leads to $3^{n}$ distinct configurations with only two tendons; in contrast, for conventional tendon-driven continuum robots, two tendons can only drive a single section with three distinct configurations (*12*). Another notable feature is that the tip of the robot can always be locally steered regardless of the shape of the remaining robot body; this enables the robot to reconfigure its shape to work at different local regions for different tasks. For instance, in Fig. 1 (K to N), with the posture already formed at the base, the tip is still capable of being steered and shortened. The complete demonstration of the testing process can be found in movie S2. Figure 1O shows the input displacement to for Fig. 1 (E and F) and Fig. 1P for Fig. 1 (H and J), demonstrating how to morph the shape of the robot by alternating the actuation sequences and magnitudes of the two inputs.

Figure 1Q illustrates the configuration of a traditional continuum robot where a total of six actuators are required to actuate three bendable sections (planar motion). In contrast, our robot can actuate an arbitrary number of sections for multibend complex shapes using only two actuators.

### Tip-everting and shape-morphing robot

**Fig. 2.**
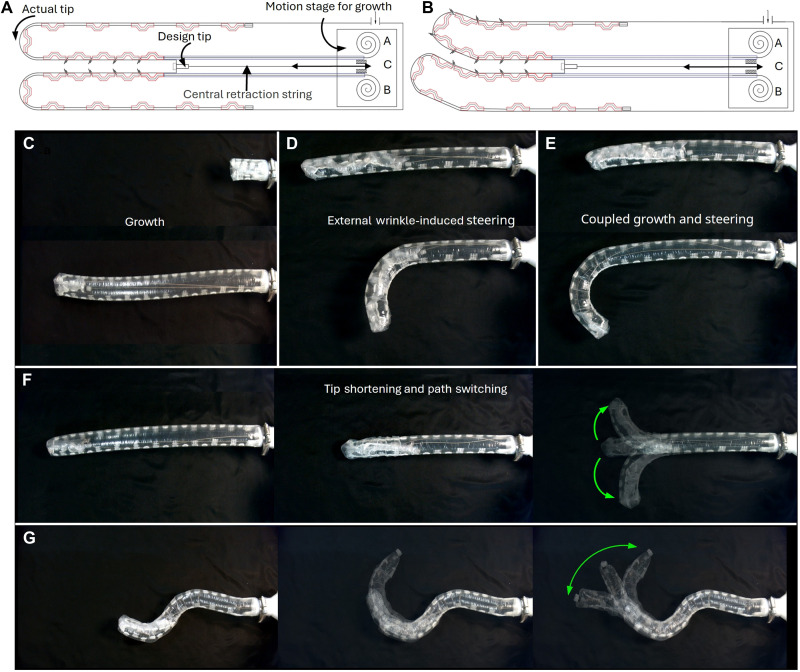
Shape-morphing and tip-growing robot. (**A**) The inflated beam is inverted to form a tip-everting robot. A central retraction string is attached to the tip to control the release and retraction of the inverted material. (**B**) Pulling one tendon propagates wrinkles outward from the design tip, while releasing it unfolds the wrinkles in the same direction. (**C**) The growth process is achieved by everting the internal material outward through high pneumatic pressure (around 2 psi). (**D**) In the external wrinkle-induced steering mode, the robot bends at the tip when a tendon is pulled to create wrinkles on the outer material of the robot, enabling bending independently of the growth process. The robot steers and grows separately in this mode. (**E**) In the coupled growth and steering mode, the robot can grow with differential lengths being released on its two sides. In this mode, the robot grows to a desired shape. (**F**) Tip shortening is achieved by simultaneously pulling both side tendons, closing the sheath gaps and shortening the robot, while allowing redirection by extending the robot again into a chosen direction. (**G**) After forming complex postures at the base, the robot can still grow and bend at the tip without affecting the base’s posture. The motion modes shown in the figure can be found in movie S3.

Inverting the inflated continuum would transform it into a tip-everting SGR. Figure 2A illustrates the design of the shape-morphing SGR. An extra central tendon is attached to the inverted material to control the release of the inner material. The location where the central tendon is attached is named as the design tip, and the most distal part of the robot is named as the actual tip. Those inverted curved sheath segments are pulled together to form a continuous sheath line along with the corresponding guiding sheath so that further tendon displacement would be transmitted to the tip of the robot for steering.

The robot can be steered via the inverted zigzag TSMs as described in the “Concepts and design principles” section. Wrinkles originate at the tip and propagate toward the base, resulting in tip-to-base steering, as shown in Fig. 2B. The SGR robot is capable of four primary motion modes: straight growth, external wrinkle-induced steering, coupled growth and steering, and tip shortening. These modes are elaborated below.

Straight growth (Fig. 2C): The robot extends forward under pneumatic pressure by everting the inner material outward from the robot’s interior. This motion forms the foundation of the SGR robot’s capabilities, enabling stable control of growth speed and distance. In this mode, the tension of the central tendon *C* is monitored to cope with the speed of tip growth.

External wrinkle-induced steering mode (Fig. 2D): The robot bends toward one side as external wrinkles propagate along its outer surface, creating a material length difference between the two sides that enables shape changes. This steering process is independent of the growth process.

Coupled growth and steering (Fig. 2E): The robot changes direction during growth due to the differential growth on the two sides. This differential growth is attributed to the different releases on the inner wrinkles by the two actuating tendons. In this mode, the pneumatic pressure is relatively high. As the robot grows and changes direction, the rear portion of the robot dynamically adjusts its shape to follow the trajectory defined by the tip, showcasing a “follow-the-leader” behavior. This capability ensures that the robot maintains a coherent and continuous path, even in complex or confined environments. This mode enables the robot to directly grow into a desired path, facilitating navigation in constrained spaces.

Tip shortening (Fig. 2F): The robot shortens its length by simultaneously pulling the tendons on both sides. By selectively releasing one or both tendons after tip shortening, it can become longer again and bend into a new orientation or resume straight growth. This capability enables the robot to grow into other interested areas.

Each motion mode uses a specific combination of tendon manipulation and pneumatic pressure control, enabling versatile operations in confined environments. The postures corresponding to these modes are depicted in Fig. 2 (C to F). The motion modes can be found in movie S3.

The robot was driven by three tendons (*A*, *B*, and *C*) and pneumatic pressure, as shown in Fig. 2 (A and B). These inputs need to coordinate one another to ensure smooth operation. For instance, relatively high pneumatic pressure (around 2 psi) is needed during growth and unbending, while corresponding tendons are relaxed to facilitate these actions. Conversely, bending and tip shortening require reduced pressure (0 to 0.5 psi) to minimize resistance, with tendons tightened to provide the necessary driving forces. In addition, by tracking the tension on each tendon, the system can automatically tighten or loosen other tendons, keeping them in a slightly pretensioned state. This automation can reduce operation complexity, prevent motion coupling among tendons, and ensure real-time responsiveness to any given maneuverers. A comprehensive overview of the drive process, including input signals, tension, and pressure variations, and the corresponding robot response can be found in Supplementary Text S1, fig. S1, and movie S4.

Thanks to the tip-to-base motion, the robot can perform various motions at the tip while keeping the shape at the base. As shown in Fig. 2G, the robot can continue to grow and bend at the tip even after the base has formed a complex shape. This ability to stably maintain its shape allows the robot to operate in complex environments without concerns about base posture changes affecting operational precision.

### Mechanics analysis

This section investigates the underlying mechanics of the inverted zigzag TSMs. First, effects of the curved sheath segments were evaluated, including their impact on force transmission. A mechanics model and experiments were developed to examine the relationship between the number of segments, characteristic angles, and friction. Next, experiments on the local buckling of the inflated beam were conducted to investigate the effects of air pressure and segment gaps on buckling behaviors. In addition, the role of friction attenuation during the bending process was analyzed.

#### Mechanics for tip-to-base sequential motion

**Fig. 3.**
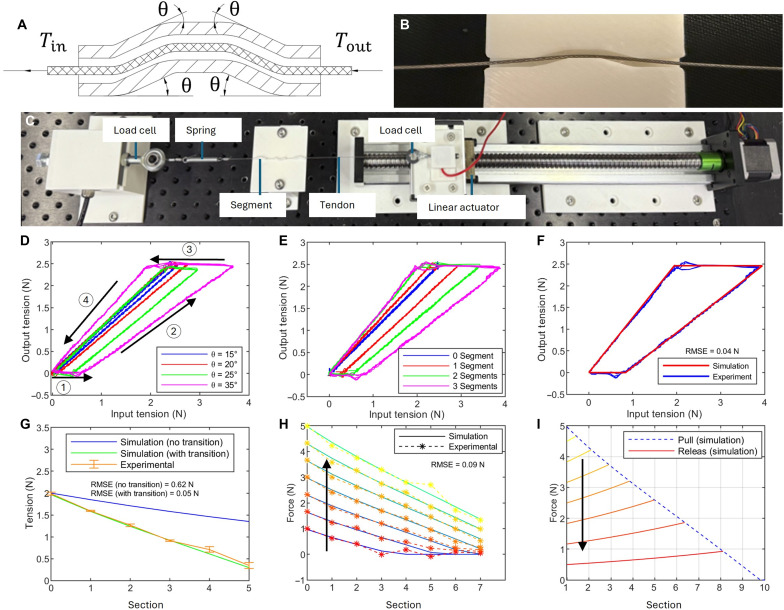
Tension transmission and friction analysis for the tendon with curved sheath segments. (**A**) A tendon goes through a curved sheath segment that has a characteristic angle θ. The tendon tension drops from $T_{\text{in}}$ to $T_{\text{out}}$ due to the friction induced by the curved angle of the sheath segment. (**B**) A breakout view of a tendon inside the curved sheath segment. (**C**) Experiment setup to measure the tensions on the two ends of the tendon with different number or types of curved sheath segments in-between. Two load cells and a linear actuator were used. (**D**) Four phases of the tension profile of the mechanism that are affected by the characteristic angle θ. Only one curved sheath segment was considered. (**E**) The tension profile with different numbers of curved sheath segment (θ = 25°). (**F**) Comparison of tension profiles in simulations and experiments. (**G**) Tension distribution along multiple sheath segments (input tension, 2 N). The model that considers the transition phase (Eq. 2) shows better accuracy. Root mean square error (RMSE) values quantify the differences between simulation and experimental results. (**H**) Tension distribution along multiple curved sheath segments during pulling (input tension increases): experiment versus simulation. This explains why the bending sequentially propagates from the tip to base during pulling. (**I**) Tension distribution along multiple curved sheath segments during releasing (input tension decreases): experiment versus simulation. This explains why the bending sequentially propagates from the tip to base during releasing.

The tip-to-base sequential motion of the SGR during loading is attributed to the decreasing tension along the tendon from the tip to the base and the snap-through force-deflection characteristics when buckling each section of the inflated beam. The decreasing tension along the tendon (0.3-mm stainless steel wire rope) is due to the purposely induced friction via a series of short and curved sheath segments (1-mm inner diameter, 3D printed with PLA) (Fig. 3, A and B). Each curved sheath segment adds an additional accumulative angle to the entire TSM, leading to additional friction to the tendon and thus tension decrease along the tendon.

A test on the curved sheath segment was conducted to verify the function of the curved sheath segment and establish a mathematical model of the relationship between the curved sheath segment and friction. The experiment setup is illustrated in Fig. 3C. The tendon was threaded through the curved sheath segments, with a motor attached to one end and a spring connected to the other. Force sensors were placed at both ends to capture tension variations along the tendon.

When the tendon is actuated, friction from the curved sheath segments progressively decreases the tendon tension from the tip to the base. This process follows the Capstan equation (*40*)

$$T_{\text{out}}=T_{\text{in}}e^{-\Phi \mu }=T_{\text{in}}e^{-4n\theta \mu }$$ (1)

where $T_{\text{out}}$ is the output tension; $T_{\text{in}}$ is the input tension; μ is the friction coefficient; Φ is the accumulative arc angle of the curved sheath segments; *n* is the number of curved sheath segments; and θ is the characteristic angle parameter of a curved sheath segment that defines how curvy is the curved sheath segment, as shown in Fig. 3 (A and B). Figure 3D shows the output-input tension profiles for a single curved sheath segment with different characteristic angles θ. In each tension profile, four phases exist: ② pull, ③ pull-to-release transition, ④ release, and ① release-to-pull transition. As observed, a larger θ would lead to a longer release-to-pull phase, indicating the need for a larger input tension for the tension to be transmitted to the output end; moreover, a larger θ would also decrease the slope of the line in the pull phase, indicating higher friction and a larger difference between the output force and input force.

The arc angle θ is critical for the motion behavior of the robot. If θ is zero, then the curved sheath segment is straight and the robot buckles at random sections with arbitrary orders, leading to uncontrollable and unpredictable motion. If θ is too large, then the force required to bend the robot would be too large, leading to excessive wear to the tendon and robot body. Thus, it is important to select θ so that the friction is sufficient to ensure the desired tip-to-base motion, which is more predictable than random buckling occurring at arbitrary sections while not imposing excessive load on the motor and tendon. In this study, four different θ angles were tested on the robot: 15°, 20°, 25°, and 35°. Figure 3D shows the hysteresis profiles of a TSM with a single curved sheath segment of the four different angles. In the pulling phase, for a given input force, the larger θ is, the higher the friction, and the smaller the output force. When θ is 15° or 20°, the curved sheath segments do not generate sufficient friction to achieve the tip-to-base motion behavior, resulting in buckling at random positions on the robot body. When θ is 25° or 35°, the robot has the desired sequential tip-to-base motion. However, the required input force when θ is 35° is much higher than that when θ is 25°. Thus, θ was selected to be 25° in this study. The friction coefficient was then identified to be 0.045 based on the above capstan equation and the experiment data shown in Fig. 3D. Note that the angle θ in this study was selected for the specific materials and geometries of the curved sheath segments and the tendon used in this study, and it may be different when different materials or geometries are used.

Figure 3E shows the experiment that investigates the force transmission when the tendon goes through multiple curved sheath segments. As can be seen, similar to the effects of arc angles, having more curved sheath segments results in a longer release-to-pull transition and a smaller slope in the pull phase, which both affect the tension transmission along the tendon. It can be seen that both the release-to-pull transition phase and the pulling phase lead to the decay of tension along the tendon, thereby increasing the force differential across curved sheath segments and ultimately enabling the tip-to-base sequential bending of the robot. A modified Capstan model was proposed to capture this force transmission behavior

$$\begin{array}{c} T_{\text{out}}=\left\{\begin{array}{cc} 0 & \text{if}\;T_{\text{in}}<T_{p}\left(\Phi \right)\\ \left[T_{\text{in}}-T_{p}\left(\Phi \right)\right]e^{-\mu \Phi } & \text{if}\;T_{\text{in}}>T_{p}\left(\Phi \right) \end{array}\right. \end{array}$$ (2)

Here, $T_{p}\left(\Phi \right)$ denotes the input force threshold required to complete the release-to-pull transition phase. To establish the relationship between $T_{p}\left(\Phi \right)$ and the arc angle Φ , a series of experiments was first conducted on even-numbered curved sheath segments (2 to 12), and the data were used to fit the corresponding relationship. The expression of $T_{p}\left(\Phi \right)$ can be found in Supplementary Text S2. Subsequently, a configuration of 1 to 12 curved sheath segments was tested and compared with simulation results(show in Fig. 3F) to validate the fitted model. More results of the 12-section tests are included in fig. S2. The experiments were conducted up to 12 segments, but similar approach can be adapted for more sections. The results in Fig. 3G further validate the modified Capstan model (Eq. 2) by comparing its predicted results with both the experimental data and the results of the Capstan model (Eq. 1). The improved model better captures the transition phase introduced by the current design, as evidenced by its closer alignment with the experimental results. Figure 3H illustrates the force decay across multiple curved sheath segments during tendon pulling (input tension increases). As the input tension increases, the transmission of the tension gradually extends to more curved sheath segments, leading to the sequential bending of these sections from the tip to the base. To quantify the model accuracy, the root mean square error (RMSE) between the simulation predictions and the experimental results was calculated. As shown in Fig. 3 (F to H), the RMSE values for the modified Capstan model (Eq. 2) remained below 0.1 N across all cases, whereas the RMSE for the original Capstan model (Eq. 1) reached ~0.6 N. These results validate the effectiveness of the modified model in more accurately capturing the friction-induced tension transmission behavior.

Figure 3I illustrates the tension distribution during tendon releasing (input tension decreases). As the input tension decreases, the drop gradually propagates along the tendon from the tip to the base, resulting in the sequential tip-to-base motion during the release phase.

#### Analysis of buckling and sequential motion

To examine the buckling dynamics and sequential motion behavior of the robot, experiments were conducted under various conditions. These experiments demonstrate the critical aspects of force dynamics, structural response, and the effects of the guiding sheath and air pressure on buckling.

**Fig. 4.**
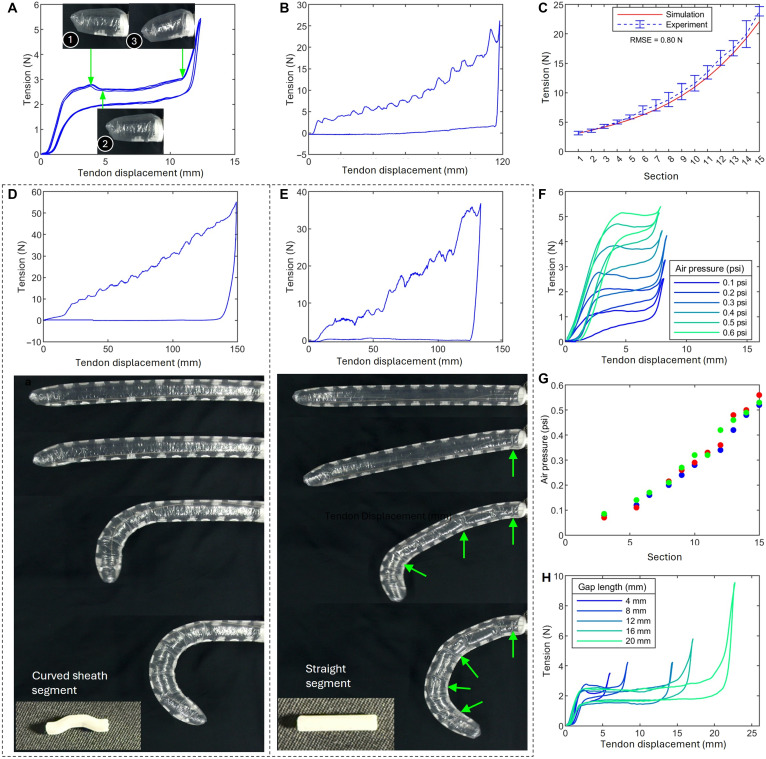
Buckling process and tension distribution and analysis. (**A**) Tension-displacement profile for robot body with single sheath gap. (**B**) Tension-displacement profile for robot body with 15 sheath gaps. The closure of each gap (local buckling of the inflated beam) causes a tension spike. (**C**) Critical input tension for the sequential closure of each gap along the robot body: experiment versus theoretical estimation using Eq. 2. (**D**) Tension-displacement profile and bending process with a guiding sheath and curved sheath segments. The robot bends sequentially from the tip to the base section by section. (**E**) Buckling with a guiding sheath and straight sheath segments: Tension increases irregularly. The robot bends at random sections, which is unpredictable. See details in movie S5. (**F**) Effect of air pressure (0.1 to 0.6 psi): Buckling tension increases linearly with pressure, rising by 1 N for every 0.1-psi increase. (**G**) Critical pressure to open each section gap during the unbending process (tendon releasing). (**H**) Effect of sheath gap length (4 to 20 mm): The gap length affects buckling duration, but not the critical tension.

To minimize external interference, initial experiments shown in Fig. 4 (A and B) were conducted without the guiding sheath. This setup allowed for the assessment of the baseline force required to initiate buckling in each curved sheath segment.

In a complete gap closing process, the tension first reaches critical value (Fig. 4A①), and then a wrinkle appears (Fig. 4A②), leading to a decrease of the input tension; then, the tension remains low until the gap is completely closed (Fig. 4A③). After that, the tension will rapidly increase because the two curved sheath segments get into direct contact.

As shown in Fig. 4A, the critical force $T_{c}$ required for a single curved sheath segment to buckle is ~2.7 N (air pressure, 0.3 psi). This threshold represents the minimum input tension to buckle one single section. Figure 4B shows the result of a similar test on a robot body with 15 curved sheath segments. It can be seen that the force gradually increases step by step (with spikes) during the tip-to-base motion. Each peak corresponds to the buckling of a section, and, by counting the number of spikes, it is possible to estimate which section is undergoing buckling.

Once the critical buckling force $T_{c}$ was determined, it was incorporated into the tension distribution model Eq. 2 to calculate the input force required for each buckling curved sheath segment. Figure 4C presents the experimentally measured input forces and their comparison with simulation results based on the friction model. The close fit between experimental and simulated data, with an RMSE of only 0.8 N, verifies the model’s accuracy in estimating the buckling forces across curved sheath segments. This result also indicates that the growth trend of the input tension follows Eq. 2, meaning that the tension required for buckling increases exponentially with the number of segments; this trend of increase ultimately limits the number of segments depending on the maximum force that can be delivered by the motor and tendons.

In practical robot implementation, an additional flexible guiding sheath was introduced at the tip (Fig. 1A) to support controlled bending from the tip to the base. This modification, as shown in Fig. 4D, increases the input force required for buckling (compare with Fig. 4B). In addition, comparative tests in Fig. 4 (D and E) demonstrate that the design with straight sheath segments (θ = 0°) buckles at random locations (unpredictable) of the inflated beam with irregular spikes in the tension-displacement profiles, whereas the design with curved sheath segments (θ = 25°) has a predictable tip-to-base bending process with regular spikes in the tension-displacement profile. The presence of curvature in the curved sheath segments provides sufficient friction, creating sufficient tension differences between adjacent sections and causing the tension in the tendon to decrease in an orderly manner from tip to base. This leads to sequential tip-to-base bending. In contrast, for Fig. 4E, the straight segments fail to provide enough friction, resulting in a lack of distinct differences between segments and ultimately causing chaotic closure without a clear sequence. This comparative process can be found in movie S5.

Internal air pressure also plays a key role in both the bending and unbending processes. As shown in Fig. 4F, during bending, the relationship between air pressure and critical buckling force is approximately linear; an increase of 0.1 psi in air pressure leads to an increase of about 1 N in buckling force. If the air pressure is too small, then the structural integrity of the robot will be affected, whereas an excessive air pressure increases the input tension on the motor and tendon. In this study, 0.3 psi during bending was selected. During the unbending process, a certain level of air pressure is required to unbend the bended sections (and to overcome the friction on the tendon). Figure 4G shows the minimum air pressure required, depending on the number of sections to be unbended during the process.

The effect of the gap length was also tested. The results in Fig. 4H indicate that the gap length has no notable effect on the critical buckling tension but primarily lengthens the buckling process itself, as a larger gap requires a larger tendon displacement for completely close the gap between the two adjacent curved sheaths.

### Kinematics analysis

In this study, a kinematics model was theoretically derived to capture the unique tip-to-base motion of the robot. The model maps the actuation space (tendon displacement input) to the configuration space (bending angles and section lengths of each section) and ultimately to the task space (tip poses). The model was then experimentally verified.

**Fig. 5.**
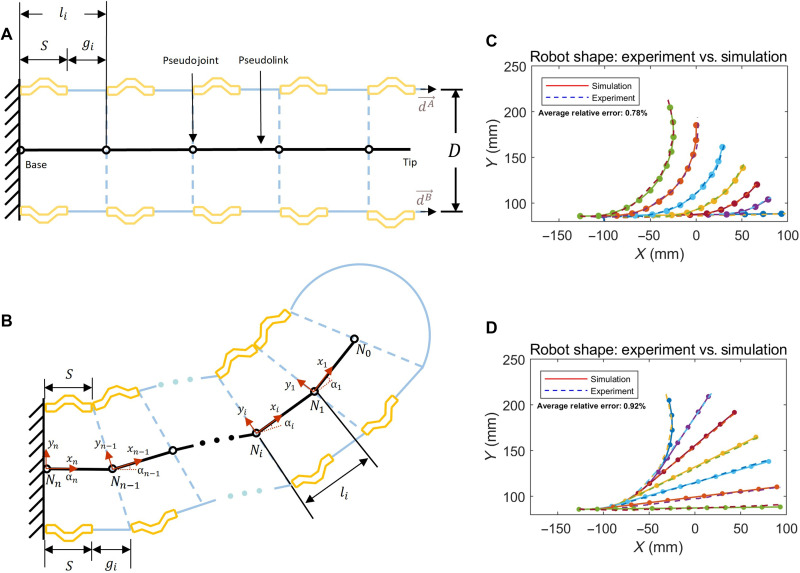
Kinematics modeling and experimental verifications. (**A**) Section discretization of the robot body. (**B**) Coordinate frames and an example robot configuration when tendon *A* is pulled. (**C**) Comparison of simulation and experiment results of the robot shape (11 sections) when tendon *A* is pulled. (**D**) The comparison when tendon *A* was subsequently released.

The robot is first discretized into *n* serially connected sections, where each section can be viewed as having a base plane, an upper plane, the robot body, and two lateral tendons encased in the curved sheaths symmetrically positioned on either side (Fig. 5A). Two adjacent sections can rotate with respect to each other if the tendon is pulled to close the corresponding gap between the adjacent sheaths.

The total length of each side of the section *i* is $S+g_{i}$ , where *S* is the length of the curved sheath, $g_{i}$ is the length of the gap between two adjacent sheaths, and i=1,2,…,n represents sections from the tip to the base. Note that, to aid the description of the tip-to-base motion, we purposely define the tip section as section 1 and the base section as section *n*. It is also worth to note that *S* is fixed, but $g_{i}\in \left[0,G\right]$ , where $g_{i}=0$ when the sheath gap is fully closed due to tendon pull and $g_{i}=G$ when the sheath gap is fully open (no wrinkle at section *i*). The change of $g_{i}$ leads to both length change and bending of section *i*.

We modified the standard pseudo-rigid-body model (*41*) to capture the unique tip-to-base motion pattern of these sections. These sections are modeled as a series of virtual links connected with virtual rotating joints (torsional stiffness is neglected for kinematics analysis). Initially, the section length is $l_{i}=S+G$ , and the rotation angles of the pivot joints are $\alpha_{i}=0$ . Once one tendon is pulled, the robot bends from the tip to the base sequentially section by section. For a given input displacement $d_{\text{pull}}$ from either tendon *A* or tendon *B*, we can determine the number of sections that would fully bent: $n_{b}={\left[\frac{d_{\text{pull}}}{G}\right]}_{\text{floor}}$ . One additional section would partially bend if $d_{\text{pull}}$ is not divisible by *G*, and the length of its corresponding side is $S+G-\left(d_{\text{pull}}-n_{b}\times G\right)$ . Each bent section features the rotation of the corresponding pivot joint and a shortened length because the sheath gap is reduced. Therefore, the length and bending angle of section *i* can be defined as follows

$$\begin{array}{c} \begin{array}{c} l_{i}=\left\{\begin{array}{cc} S+G/2, & \;i\in \left[1,n_{b}\right]\\ S+G-\left(d_{\text{pull}}-n_{b}\times G\right)/2, & \;i=n_{b}+1\\ S+G, & \;i\in \left[n_{b}+2,n\right],n_{b}<n-1 \end{array}\right. \end{array} \end{array}$$ (3)

$$\begin{array}{c} |\alpha_{i}|=\left\{\begin{array}{cc} G/D, & \;i\in \left[1,n_{b}\right]\\ \left(d_{\text{pull}}-n_{b}\times G\right)/D, & \;i=n_{b}+1\\ 0, & \;i\in \left[n_{b}+2,n\right],n_{b}<n-1 \end{array}\right. \end{array}$$ (4)

where $i\in \left[n_{b}+2,n\right]$ represents the sections that are not bent, i.e., straight, noting that *n* represents the total number of all the sections; $i\in \left[1,n_{b}\right]$ represents the sections that are fully bent, i.e., the sheath gap on the corresponding side of the section is fully closed; $i=n_{b}+1$ represents the partially bent section (not fully bent) if $d_{\text{pull}}$ is not divisible by *G*; and *D* is the diameter of the inflated beam. Here, $|\alpha_{i}|$ represents the absolute magnitude of $\alpha_{i}$ , where $\alpha_{i}\geq 0$ when $d_{\text{pull}}$ is from tendon *A*, and $\alpha_{i}\leq 0$ when $d_{\text{pull}}$ from tendon *B* based on the coordinate system defined in (Fig. 5B). Note that $\alpha_{i}$ determines the achievable curvature of the robot, and it can be tailored for different curvature requirements by adjusting *G* and *D*.

Subsequently, if the same tendon is released (no more than the pulled displacement), then the bent sections of the robot would unbend to become straight section by section from the tip to the base. The number of unbend sections is $n_{\text{ub}}={\left[\frac{d_{\text{release}}}{G}\right]}_{\text{floor}}$ , with $d_{\text{release}}$ representing the tendon displacement released; as such, the lengths and bending angles of the affected sections should be updated as follows

$$\begin{array}{c} l_{i}=\left\{\begin{array}{cc} S+G, & i\in \left[1,n_{\text{ub}}\right]\\ S+G/2+\left(d_{\text{release}}-n_{\text{ub}}\times G\right)/2, & i=n_{\text{ub}}+1 \end{array}\right. \end{array}$$ (5)

$$\begin{array}{c} |\alpha_{i}|=\left\{\begin{array}{cc} 0, & i\in \left[1,n_{\text{ub}}\right]\\ \left[G-\left(d_{\text{release}}-n_{\text{ub}}\times G\right)\right]/D, & i=n_{\text{ub}}+1 \end{array}\right. \end{array}$$ (6)

where $i\in \left[1,n_{\text{ub}}\right]$ represents the sections restored to their initial straight status and $i=n_{\text{ub}}+1$ represents the section that is partially unbending (not fully straight) if $d_{\text{release}}$ is not divisible by *G*. All other sections are unaffected, keeping their statuses as defined by Eq. 4. Note that the sign of $\alpha_{i}$ in Eq. 6 is the same as that in Eq. 4.

For more complex actuation sequences, the length and angle of the sections can be derived by repetitively using Eqs. 3 to 6 based on the order of the actuation sequences.

Once the lengths and rotation angles of all sections are calculated, the homogeneous transformation matrix approach is used to calculate the positions of the link node in the global coordinate frame, representing by ${\left[x_{i},y_{i},1\right]}^{T}$

$$\begin{array}{c} {\left[\begin{array}{c} x_{i}\\ y_{i}\\ 1 \end{array}\right]}_{{\text{frame}}_{n}}=T_{n-1}^{n}T_{n-2}^{n-1}\cdots T_{i}^{i+1}{\left[\begin{array}{c} l_{i}\\ 0\\ 1 \end{array}\right]}_{{\text{frame}}_{i}},\;i=0,1,\;\ldots ,n \end{array}$$ (7)

Here, $T_{i}^{i+1}$ represents the transformation matrix to project a node in ${\text{frame}}_{i}$ to ${\text{frame}}_{i+1}$ , including the relative rotation and translation of these two frames

$$T_{i}^{i+1}=\left[\begin{array}{cc} R_{i} & t_{i}\\ 0 & 1 \end{array}\right]=\left[\begin{array}{ccc} \text{cos}\alpha_{i} & -\text{sin}\alpha_{i} & l_{i+1}\\ \text{sin}\alpha_{i} & \text{cos}\alpha_{i} & 0\\ 0 & 0 & 1 \end{array}\right]$$ (8)

Note that the proposed modified pseudo-rigid-body model is different from the widely used conventional constant-curvature model (*11*, *42*) of continuum robots in two aspects: (i) the former models the tip-to-base motion pattern of the proposed robot, while the latter assumes constant curvature regardless of actuation; (ii) the former considers the length changes of the robot’s sections during bending or unbending while the latter does not.

To validate this kinematics model, a testing platform shown in fig. S3 was developed to compare the estimated and actual shapes of an 11-section robot during actuation. The input displacement and robot’s shapes during actuation were recorded. The input displacement was also fed into Eqs. 3 to 8 to estimate the shapes of the robot that are represented by the coordinates of the nodes 0 to *n*. The experiment and simulation results during the bending and unbending process are shown in Fig. 5 (C and D), and full comparisons are shown in movie S6. The average relative errors for all the shapes during the bending and unbending processes were 0.78 and 0.92%, respectively. The relative error for each shape is calculated as the average distance from the nodes in simulation to the corresponding shape in the experiment, divided by the robot length. This confirms the accuracy of the kinematics model in estimating the robot’s shape with its unique tip-to-base motion during the bending and unbending processes.

#### Reachable workspace analysis

**Fig. 6.**
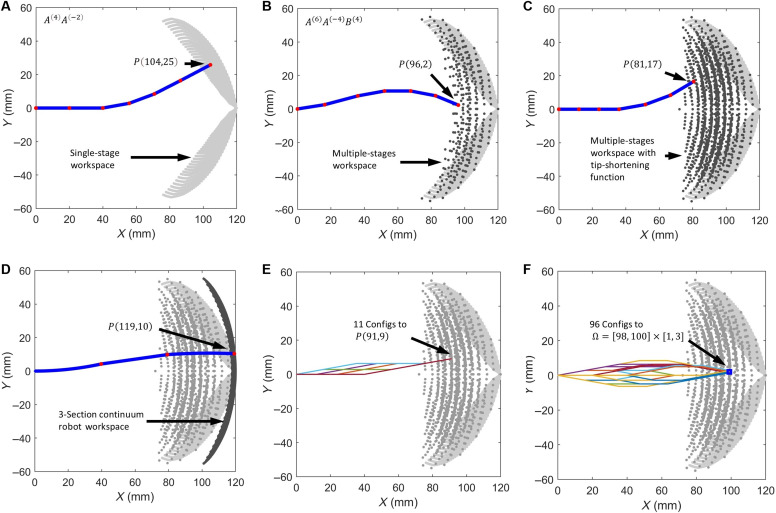
Kinematic workspace expansion and comparison with various different configurations of a six-section HasMorph robot. (**A**) The workspace achieved with a single bending stage, i.e., a single tendon pull/release cycle. (**B**) The expanded workspace when multiple bending stages (multiple pull/release cycles) are incorporated. (**C**) The workspace was further expanded by allowing the two sheath gaps of one section to close, i.e., the section is straight but its length shortens from *S* + *G* to *S*. (**D**) Comparison of the workspace of the proposed robot (two tendons required) with that of a conventional three-section tendon-driven continuum robot (six tendons required), highlighting the proposed robot’s superior coverage thanks to its multistage sequential bending and shortening mechanisms. (**E**) The robot reaches the same target position with 11 different configurations using a combination of shortening and multistage bending strategies. (**F**) The robot have 96 different configurations to reach the blue square area Ω.

The experimentally verified kinematics model (Eqs. 3 to 8) was then used to analyze the workspace of the robot based on the Monte Carlo method under various displacement input configurations. Figure 6 (A to C) presents the workspace of an example of the shape-morphing robot (six sections) computed using the kinematics model. In Fig. 6A, the shaded region illustrates the workspace generated by single-stage motion, a single tendon pull/release cycle. For instance, to make the tip $N_{0}$ reaching the point P(104,25) , tendon *A* is pulled to bend the first four sections of the robot and then released to unbend the first two sections. It is clear that, due to hysteresis in the pull and release phases, the robot can reach a two-dimensional area via bending. Figure 6B shows an expanded workspace via multistage motion, i.e., multiple pull/release cycles. For example, pulling tendon *B* to bend two tip sections after pulling/releasing tendon *A* extends the robot’s tip into previously unreachable areas of the workspace. Note that the void space among those dots can be reached by the robot but is not included in the simulation to reduce computation power. Figure 6C highlights the effect of the shortening mechanism, where some sections become shorter because of the shortening of their both sides (e.g., tendons *A* and *B* are both pulled). This mechanism substantially increases the workspace, allowing the robot tip link node to reach more area. For instance, to make $N_{0}$ reaching point P(81,17) , the robot shortens all sections before extending specific segments to their desired lengths. Figure 6D compares the workspace of the proposed robot with that of a conventional three-section continuum robot driven by six tendons shown in Fig. 1Q. The workspace of the conventional continuum robot was simulated using the widely used constant-curvature kinematics model (*43*, *44*). The results show that the proposed robot offers a substantially larger and more adaptable workspace than the conventional continuum robot. Figure 6E shows that the robot can reach the same target position P(91,9) with multiple configurations, demonstrating the dexterity of the robot. Last, Fig. 6F shows that the robot can have 96 different configurations to reach the 4-mm^2^ area Ω=x[98,100]×y[1,3] , demonstrating the robot’s capability of reaching target areas with multiple configurations and execution strategies.

### Experimental validation of applications

**Fig. 7.**
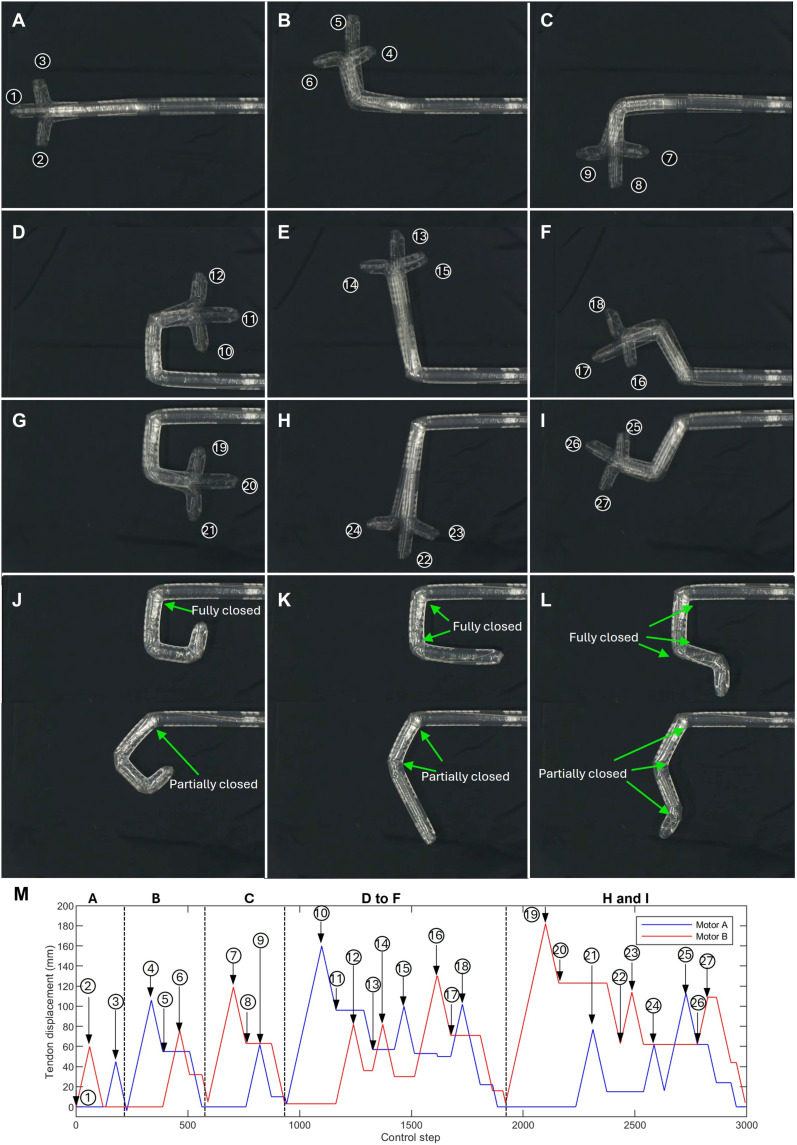
Shape-morphing states demonstrated by three-section robot. To enhance the visual clarity of each configuration, a three-section robot was used, with segment lengths of 42, 72, 102, and 132 mm and uniform gap length of 60 mm, each inducing a 90° bending when fully closed. (**A** to **I**) The robot exhibits 27 distinct configurations with three sections (i.e., $3^{n}$ configurations, as explained in the “Concepts and design principles” section). (**J** to **L**) Comparison between partially closed and fully closed gap states. Even when the gap is only partially closed, the resulting configurations can be maintained without the need for additional locking mechanisms, illustrating the inherent stability and wide coverage of the configuration space of the robot. (**M**) Motor control sequence corresponding to the configurations shown in (A) to (I). See movie S7 for the full demonstration.

To quantitatively demonstrate shape morphing, we made a three-section robot actuated by two tendons following the HasMorph concept. Each section of the robot can bend about 90° maximum. Even with only two tendons, this three-section robot can have 27 (i.e., $3^{n}$ , as explained in the “Concepts and design principles” section) distinct configurations, depending on the actuation sequences illustrated in Table 1. In contrast to the traditional three-section continuum robot shown in Fig. 1Q, which requires six tendons to achieve full control, the proposed HasMorph concept and inverted zigzag TSM lead to exponential configuration expansion even only two tendons. This advantage becomes increasingly notable as the number of sections increases. Note that the 27 distinct configurations in Fig. 7 (A to I) only include the configurations where the sections are bent into their extreme angles; more configurations can be obtained if the sections are bent into intermediate angles (Fig. 7, J to L). Figure 7 (J to L) compares postures resulting from fully (extreme angles) and partially (intermediate angles) closed gaps. Even when the gaps are not fully closed, the corresponding configurations remain stable without any additional locking mechanisms or active adjustments. This ability greatly expands the robot’s reachable workspace, enabling it to access arbitrary regions rather than being constrained to discrete curves or predefined trajectories.

**Table 1.** Configurations and their corresponding motor inputs. For each configuration, the actuation sequence is denoted by $A^{\left(n\right)}$ and $B^{\left(n\right)}$ , indicating that motors A or B pull or release the tendons of the first n sections.

Configuration	Input	Configuration	Input	Configuration	Input
①	–	②	$B^{\left(1\right)}$	③	$A^{\left(1\right)}$
④	$A^{\left(2\right)}$	⑤	$A^{\left(2\right)}A^{\left(-1\right)}$	⑥	$A^{\left(2\right)}A^{\left(-1\right)}B^{\left(1\right)}$
⑦	$B^{\left(2\right)}$	⑧	$B^{\left(2\right)}B^{\left(-1\right)}$	⑨	$B^{\left(2\right)}B^{\left(-1\right)}A^{\left(1\right)}$
⑩	$A^{\left(3\right)}$	⑪	$A^{\left(3\right)}A^{\left(-1\right)}$	⑫	$A^{\left(3\right)}A^{\left(-1\right)}B^{\left(1\right)}$
⑬	$A^{\left(3\right)}A^{\left(-2\right)}$	⑭	$A^{\left(3\right)}A^{\left(-2\right)}B^{\left(1\right)}$	⑮	$A^{\left(3\right)}A^{\left(-2\right)}A^{\left(1\right)}$
⑯	$A^{\left(3\right)}A^{\left(-2\right)}B^{\left(2\right)}$	⑰	$A^{\left(3\right)}A^{\left(-2\right)}B^{\left(2\right)}B^{\left(-1\right)}$	⑱	$A^{\left(3\right)}A^{\left(-2\right)}B^{\left(2\right)}B^{\left(-1\right)}A^{\left(1\right)}$
⑲	$B^{\left(3\right)}$	⑳	$B^{\left(3\right)}B^{\left(-1\right)}$	㉑	$B^{\left(3\right)}B^{\left(-1\right)}A^{\left(1\right)}$
㉒	$B^{\left(3\right)}B^{\left(-2\right)}$	㉓	$B^{\left(3\right)}B^{\left(-2\right)}B^{\left(1\right)}$	㉔	$B^{\left(3\right)}B^{\left(-2\right)}A^{\left(1\right)}$
㉕	$B^{\left(3\right)}B^{\left(-2\right)}A^{\left(2\right)}$	㉖	$B^{\left(3\right)}B^{\left(-2\right)}A^{\left(2\right)}A^{\left(-1\right)}$	㉗	$B^{\left(3\right)}B^{\left(-2\right)}A^{\left(2\right)}A^{\left(-1\right)}B^{\left(1\right)}$

Figure 7M presents the complete control sequence corresponding to the configurations in Fig. 7 (A to I). Through alternating sequential inputs, the robot achieves a wide range of shape-morphing behaviors using only two motors. A full experiment demonstration of all configurations and control sequences is provided in movie S7.

Table 1 lists the specific motor inputs corresponding to each configuration, expressed in terms of $A^{\left(n\right)}$ and $B^{\left(n\right)}$ , indicating that motors A or B actuate (pull or release) the tendons of the first n segments. Notably, for the same input values, entirely different shapes can be obtained; this is due to the variation in actuation sequence. For instance, configurations ⑥, ⑨, ⑬, ⑰, ㉔, and ㉖ all involve pulling one segment (~60 mm) with both motors, yet exhibit distinct postures. This exemplifies one of the key advantages of harnessing hysteresis: The actuation sequence directly influences the final shape, notably reducing the need for additional actuators.

**Fig. 8.**
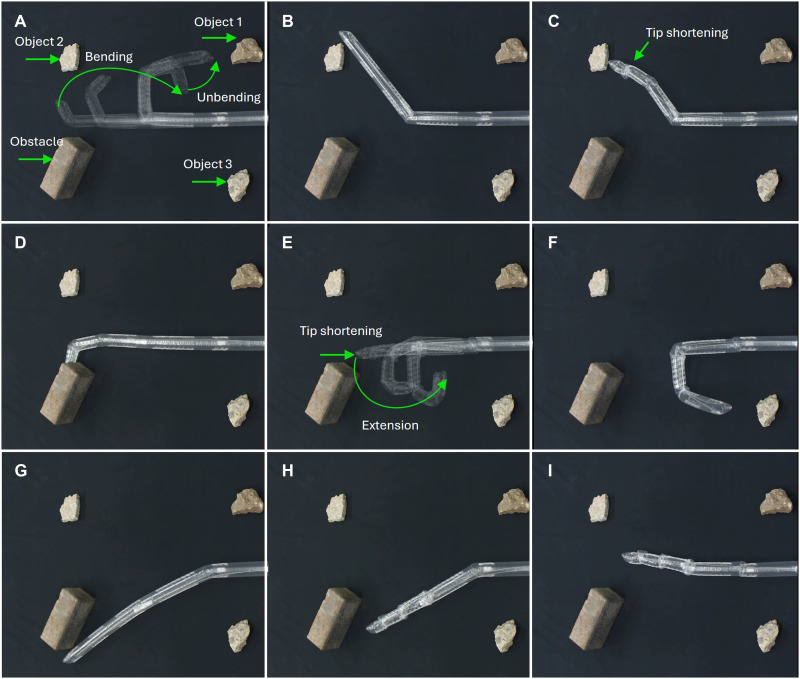
Tip shortening demonstrated with a three-section robot. By pulling both tendons simultaneously, the sheath gaps on both sides of the robot close sequentially from the tip, resulting in shortening in length. (**A**) The robot approaches the object 1 through a bending followed by unbending. (**B**) The object 2 obstructs the robot’s return. (**C**) Tip shortening is used to reach the object 2. (**D**) The robot is blocked by an obstacle. (**E** and **F**) The robot bypasses the obstacle and reaches the object 3 through shortening tip followed by directed extension. (**G**) The robot is obstructed while attempting to return along the original shape. (**H** and **I**) The robot shortens to bypass the obstacle and returns to its original shape. See movie S8 for the full demonstration.

The robot is capable of reaching target objects through shape morphing and tip shortening, as demonstrated in Fig. 8A. The ability to shorten its tip provides high adaptability to varying environments and notably expands the robot’s effective workspace. The robot can also access the object by shortening its tip, as shown in Fig. 8 (B and C).

In the presence of environmental obstacles, the combination of shortening and regrowth allows the robot to alter its motion path to reach target positions or return to its initial state while avoiding collisions (Fig. 8, D to I). Tip shortening not only increases the reachable area but also enables multiple trajectory options to the same target. This enhances the robot’s operational flexibility and reduces the need for complex path planning. The complete experimental demonstration of these capabilities is provided in movie S8.

**Fig. 9.**
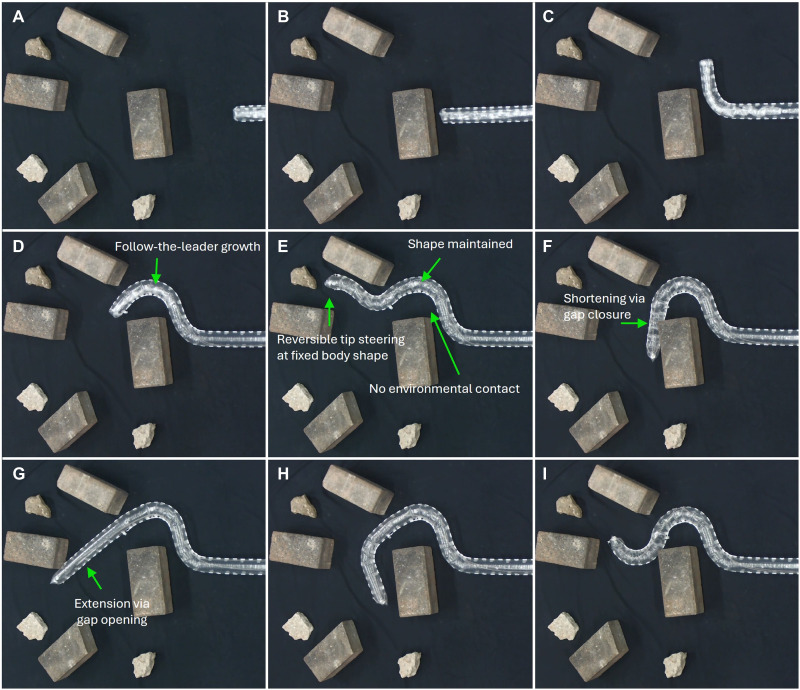
A 1.2-m robot navigates in a cluttered environment via follow-the-leader tip growth, reversible steering, and tip shortening. To enable shape-morphing operation at this length, the segment gap was increased to 20 mm, and three straight sheath segments were inserted between two curved sheath segments to serve as low-friction structural supports. Additionally, the sheath segments were arranged along the robot’s exterior to facilitate smoother growth. (**A**) The robot begins in its default configuration. (**B**) The robot begins to grow. (**C** and **D**) The robot navigates around the obstacles via follow-the-leader growth, forming an “S” shape. (**E**) The robot grows and performs reversible steering to approach the target object while maintaining its complex body shape without relying on contact with the environment. (**F**) The robot becomes shorter to return to another branching area on the other side of the obstacle. This was achieved by pulling the two tendons simultaneously. (**G** and **H**) The robot grows by releasing tendons to reopen the sheath gap, continuing the exploration in a new direction and approaching other two additional target objects. (**I**) Based on the release and reconstruction of the previously formed bends, the robot reapproaches the initial target object. The corresponding demonstration is provided in movie S9.

To reduce cumulative friction and extend the robot’s effective range, the robot structure was modified by increasing the gap length and inserting straight segments between curved segments and routing the tendon sheath segments along the robot’s exterior. As shown in Fig. 9, a 1.2-m-long robot was made to adaptively explore an unstructured, confined environment with obstacles.

In Fig. 9 (A to E), the robot undergoes growth and performs four bends to navigate around obstacles and ultimately reach the location of the target object. In Fig. 9C, the robot first performs a bend to avoid the obstacle. In Fig. 9D, it exhibits follow-the-leader growth by continuing to grow in a curved posture, fully crossing over the obstacle. It then proceeds with a reverse bend and further growth to reach the target region, as shown in Fig. 9E. Throughout this process, the robot adaptively changes its growth direction and forms multibend complex body shapes to avoid and conform to the obstacles (without direct contact with the obstacles) while reversibly steering and extending the tip. Note that these body shapes were not prescribed in the design of the robot.

In Fig. 9F, the robot shortens its tip to return to a previous branching point. The enlarged gap design allows for more effective retraction over a longer distance. Following tip shortening, the robot reopens the intersegment gap and extends its body length, enabling it to approach two additional target objects along a new direction of exploration (Fig. 9, G to H). In addition, the robot can undo previously formed bends and reconfigure its shape to reapproach the initial target object (Fig. 9I). The complete motion process is shown in movie S9.

This sequence of operations not only demonstrates the robot’s ability to perform reliably at extended structural lengths but also highlights the key advantages of the HasMorph strategy: seamless integration between shape morphing and tip growth, highly dexterous and reversible bending, tip steering without affecting the body shape, the ability to lock and retain previously formed complex configurations, and controllable tip shortening and extension. These features collectively enable dexterous exploration with soft growing robots.

## DISCUSSION

Our study demonstrates that (i) the HasMorp concept can leverage shape hysteresis to achieve reversible, multisectional bending of soft continuum robots by alternating the actuation sequences of only two tendon actuators, notably increasing the configuration space and workspace of the robot. (ii) The inverted zigzag TSM actuation mechanism can lead to shape hysteresis of the robot by purposely inducing friction to the tendon routing, mechanically fulfilling the HasMorp concept. (iii) HasMorph is also suitable for SGRs and provides an effective way to controlling the growth direction as well as shape formation of the robot for navigation in cluttered environment.

Traditionally, hysteresis in robotics has been regarded as a limitation, often leading to actuation or sensing errors that require compensation or elimination (*36*, *37*, *39*). In contrast, this work leverages shape hysteresis to achieve reversible, multisectional shape transformations by alternating the actuation sequences of tendons. This provides a unique perspective, approach, and application for using hysteresis. In addition, unlike conventional passive underactuation systems that rely on uncertain environmental interactions (*15*, *16*), HasMorph can actively control the bending of each section through a sequence of actuation’s TSM, enabling complex shape transformation and locking.

Many existing SGR designs are limited to producing a single bend at any given time due to their length-varying nature (*22*, *24*), notably restricting their dexterity and movement capabilities. While some approaches achieve complex multibend configurations, they often rely on bulky hardware or numerous actuators (e.g., valves and tubes) (*45*), while others are constrained by irreversible steering or predefined shapes (*20*, *30*, *31*). HasMorph overcomes these challenges through a compact inverted zigzag TSM, enabling multisectional, reversible shape morphing and follow-the-leader tip growth.

Traditional straight or helical tendon routings (*25*, *30*, *31*) for continuum robots can only generate three distinct configurations (positive/negative bends and straight) with two tendons. To increase the configuration space, a variable-stiffness body is needed (*30*), which complicates the design and control of the robot. Multistable metamaterials or structures (*46*–*48*) can be used to reconfigure a robot with more configurations, but they require many multistable modules and are still limited in configurations. While shape-memory alloys exhibit intrinsic hysteresis (*49*), their transformations are limited to predefined shapes. However, as demonstrated, HasMorph can exponentially increase the configuration space with only two actuators: $3^{n}$ distinct configurations for an *n*-section robot, and *n* be easily scaled as needed (3 billion configurations for a 20-section robot).

From a mechanical design perspective, the inverted zigzag TSMs addresses several critical challenges of continuum robots. By introducing controlled friction through the inverted zigzag sheath segments, the system achieves tip-to-base sequential steering, where bending begins at the distal tip and propagates incrementally to base sections. This zigzag tendon routing design simplifies kinematics and ensures predictable motion. Otherwise, a straight tendon routing in this case would bend the robot at random locations and the motion is unpredictable (Fig. 4). A helical tendon routing (*38*) can only drive a continuum to a helix shape and the process in-between is random and unpredictable. Furthermore, the tip-to-base motion allows the robot to steer its tip without affecting the other part of the body, enabling complex shape formation and path stabilization during operation. Additionally, HasMorph integrates a tip shortening mechanism, proposing a feasible alternative to the challenge of retracting SGRs once they are deployed (*50*, *51*). By actively controlling both tendons, the robot achieves precise and reversible shortening, enabling dynamic posture adjustments during navigation and seamless path transitions. For instance, the robot can retract its tip to avoid an obstacle, backtrack along a previous route, or switch to a new path, notably improving its adaptability in confined or dynamic settings. These capabilities are critical for tasks requiring precise navigation, such as avoiding obstacles while reaching a target, as well as for path retracing, multipath exploration, and real-time shape reconfiguration.

HasMorph supports two steering modes: steering with and without growth, making it suitable for both mobile navigation and in situ operations. Steering without growth is achieved by creating external wrinkles, while steering with growth is realized by releasing internal wrinkles at the tip. This dual-mode capability further enhances the robot’s versatility, especially in dynamic environments that require rapid adaptability. For instance, this feature allows the robot to seamlessly switch between navigating through narrow spaces and performing manipulations at target locations, different from the work in (*20*) where steering and tip growth are coupled.

Unlike (*38*) where the tendon sheath mechanism is at the outer surface of the robot, we mounted the TSM inside the robot body, which effectively shields the driving mechanism from external environmental interactions. This design notably enhances the robot’s safety, durability, and reliability, reducing the risk of damaging the environments (e.g., the colon to be inspected) and the driving mechanism itself.

Experimental validations confirm the practical advantages of HasMorph. By harnessing the discrepancies between loading and unloading paths induced by hysteresis, the robot achieves precise, lockable, multishape transformations with minimal actuation. The frictional locking mechanism ensures that the robot can stabilize its configuration, adapt dynamically to environmental changes, and maintain its path without relying on complex global path planning. In maze navigation experiments, HasMorph demonstrated superior adaptability using a follow-the-leader strategy, effectively navigating narrow passages and complex junctions. These results underscore its potential applications in search-and-rescue operations, medical navigation, and industrial inspections.

Despite these advantages, there are some limitations with the current work. The exponential increase in friction with the number of curved sheath segments limits robot length, operational range, and energy efficiency. It is possible to fine-tune the parameters of the zigzag mechanism for longer robots or for specific applications, for instance, only sparsely use the zigzag mechanism to generate a few necessary rotational joint for a relatively longer link (Fig. 7). Although this study is limited to planar motion to establish a clear and rigorous foundation for this concept, extending the approach to spatial motion using the shape hysteresis principle is an exciting avenue for future work. Inverse kinematics would also be needed for the low-level control of the robot to come to a target tip pose or a desired shape. From a control perspective, further exploration of inverse kinematics models and real-time path planning strategies will help refine trajectory accuracy and enhance navigation efficiency. In addition, the stiffness of an inflated robot is mainly dependent on the air pressure, which limits its payload capacity. Future efforts will focus on optimizing the inverted zigzag sheath geometry, dynamically tuning the friction coefficient through material innovation, three-dimensional shape morphing, inverse kinematics and path planning, and variable stiffness modules for enhanced payloads when needed.

By redefining hysteresis as a functional feature and implementing it using a compact inverted zigzag TSMs, this study establishes a framework for the shape morphing of continuum robots and the emerging SGRs. The HasMorph system’s exponentially increased the robot’s configuration space, predictable tip-to-base steering, dual-mode steering capability, and internalized driving mechanism protection notably enhance its capabilities in navigating confined environments, morphing shapes on demand, and stabilizing path configurations. These findings provide a solid theoretical and practical foundation for autonomous navigation and adaptive manipulation in medical, industrial, and search-and-rescue applications.

## MATERIALS AND METHODS

### Fabrication

The design of the robot’s segments involves a modular structure to enable flexible assembly and adaptation. Each curved sheath segment has a total length of 12 mm, an outer diameter of 2 mm, an inner diameter of 1 mm, and a standard internal angle (θ) of 25°. These parameters can be adjusted according to the specific dimension requirements of the robot. The curved sheath segments were fabricated using a Bambu Lab X1 Series 3D printer with PLA as the printing material. The tendons were made from 0.3-mm steel wire, while the outer wall of the robot was constructed using a plastic film with a diameter of 34 mm.

To fabricate the robot, the segments were aligned in a straight line along the outer surface of the plastic film using adhesive tape, with a typical gap of 8 mm between adjacent segments. This gap can be adjusted according to specific design needs. After attaching segments on both sides of the film, a tendon was threaded through the aligned segments and tied into a fixed knot at the base. A long guiding sheath was then placed around the tendon to provide support and protection while also ensuring that the input force was effectively transferred to the robot’s tip. The plastic film was then inverted inward, enclosing the segments, tendons, and sheath, and the tip was sealed to complete the fabrication of the continuum robot.

To accommodate varying task requirements, the arrangement of segments can be adjusted accordingly. While the same curved segments are used, additional straight segments may be introduced as frictionless structural elements to extend the robot’s operational range or provide mechanical support. Furthermore, the gap length between segments can be modified to enable larger angular deformations as needed for specific tasks.

All curved sheath segments used throughout the experiments have a standard length of 12 mm and an internal curvature angle of 25°. The gap length between adjacent curved segments varies depending on the experimental scenario.

Figures 1, 2, and 4 and figs. S1 and S4 used robots with a gap length of 8 mm between curved sheath segments. No straight segments were introduced in these configurations.

Figures 7 and 8 used robots with extended gap lengths of 60 mm. In these cases, each functional segment consisted of one curved sheath segment connected to 2, 4, 6, or 8 straight segments, each measuring 15 mm in length, to enhance visual clarity and enable larger-scale deformation.

Figure 9 presents a robot fabricated using an external assembly strategy. Tendons and sheath segments were affixed to the outside of the robot’s plastic body using adhesive. A gap length of 20 mm was adopted, and each functional unit was constructed by attaching one curved sheath segment followed by three 12-mm straight segments.

### Growth actuation and control

For the actuation system, a sealed chamber was fabricated to house the key control components. Inside the chamber, three motors were installed: One central motor was dedicated to the central string to control growth, while the other two motors, which were mechanically attached to the rotating wheel of the central motor, controlled the bending motion of the robot. A proportional pneumatic valve was used to regulate the air pressure in the chamber.

### Friction test

To investigate the mechanical properties of the curved sheath segments and their impact on force transmission, a series of friction tests were conducted. The test bed (Fig. 3B) consisted of a motorized ball-screw linear slider that drives a tendon to pull a spring. Each end of the tendon is attached to a load cell for force measurement. The tendon also go through curved sheath segments with varying numbers of segments (0 to 15 segments) or different internal angles (ranging from 15° to 35°). The linear slider applied cyclic motion to the tendon, and the change in force at both ends was recorded.

The force attenuation, which quantifies the reduction in force transmitted along the tendon as it passes through the curved sheath segments, was analyzed and calculated. This analysis allowed for the quantification of the mechanical impact of segment configuration (i.e., number of segments and internal angle) on the force transmission.

### Buckling test

To evaluate the critical conditions under which the robot deforms, a buckling test was performed. The robot was attached to a 3D printed sealed mount, with tendons exiting through dedicated holes at the back of the mount. The tendons were connected to a linear motor via a load cell to measure the applied force. Experiments were conducted under varying conditions, including the number of segments (1 and 15), air pressure (0.1 to 0.6 psi), gap between segments (4 to 20 mm), and the presence or absence of a sheath. During the test, the linear motor cyclically pulled the tendon while the changes in the force profile were recorded to identify the onset of buckling.

### Kinematics analysis

A test bed (see fig. S3) was set up to experimentally verify the kinematics model. The test bed consists of an 11-section inverted zigzag robot body marked with a green paper line attached for shape recognition, fixed onto a 3D printed PLA pneumatic housing. A linear slider drives a tendon at a constant speed (12.5 mm/s). An overhead high-speed camera was used to capture the robot’s shape, and a checkerboard with 5 squares by 4 squares (20-mm width) was used for calibration.

In this experiment, the section length was set to *L* = 20 mm, the sheath length *S* was 12 mm, and the gap length *G* was 8 mm. The diameter of the inflated beam is *D* = 45 mm; this leads to a maximum bending angle of 10.2° for each section.

The recorded video was analyzed using sort-line tracking to extract the shape of the robot’s body during the action, as demonstrated in movie S6. The green paper line attached to the plastic robot body central was recognized and then skeletonized to represent the centerline of the robot body. The coordinates are mapped and calculated on the basis of checkerboard-based projection. Each frame of the extracted motion trajectory was subsequently compared with the predicted trajectory derived from simulations under identical displacement inputs. This analysis facilitated the evaluation of the robot’s kinematic behavior, motion accuracy, and the correlation between the experimental and simulated trajectories.

To compute the 2D workspace of the robot, a Monte Carlo simulation approach was used. The simulation model consisted a six-section inverted zigzag robot with a segment sheath length of *S* = 12 mm, a gap length of *G* = 8 mm, and a section bending limitation of 10.2°, constrained by the inflated beam’s diameter of 45 mm.

This method involves generating a large number of random section’s configurations by combining each section’s angle and coupled length. For each generated configuration, the forward kinematics model, derived from Eqs. 3 to 8, was used to compute the corresponding tip poses.

Figure 6A illustrates the bending and unbending phases of the robot under different magnitudes of input displacement [ $d_{\text{pull}}\in \left(0,n\times G\right)$ ]. The tip pose is then calculated by the forward kinematics model with the displacement. Figure 6B presents the expanded workspace with each section configuration having three possible angles (negative, 0°, and positive) with its coupled length. Only extreme values were considered in the simulation for compute efficiency. Figure 6 (C to F) presents the expanded workspace with each section having four configurations, where 0° is related to two configurations: The length of the section is either *S* or *S* + *G*.

The simulation results were aggregated to produce a comprehensive map of the robot’s 2D workspace. As shown in Fig. 6, the workspace is represented as the shaded region encompassing all reachable endpoint positions. The Monte Carlo approach effectively captures the nonlinear and hysteresis-driven behavior of the robot, providing insights into the influence of design parameters such as the tendon sheaths, shortening mechanism, and segment configuration. By comparing the results under various configurations, the simulation highlighted the notable enhancement in workspace flexibility and size achieved by the proposed design.
