# Supplementary material for: Hysteresis-assisted shape morphing for soft continuum robots
Source: Sci Adv. 2025 Oct 15;11(42):eadx3024. doi: 10.1126/sciadv.adx3024 (PMC13155642; doi:10.1126/sciadv.adx3024)
Supplement: Supplementary file 1 — Figs. S1 to S4 Supplementary Text S1 to S3 Legends for movies S1 to S11 [file sciadv.adx3024_sm.pdf]

Science Advances
AAAS

Supplementary Materials for

Hysteresis-assisted shape morphing for soft continuum robots

Zheyuan Bi *et al.*

Corresponding author: Lin Cao, l.cao@sheffield.ac.uk

Sci. Adv. **11**, eadx3024 (2025)
DOI: 10.1126/sciadv.adx3024

The PDF file includes:

Figs. S1 to S4
Supplementary Text S1 to S3
Legends for movies S1 to S11

Other Supplementary Material for this manuscript includes the following:

Movies S1 to S11

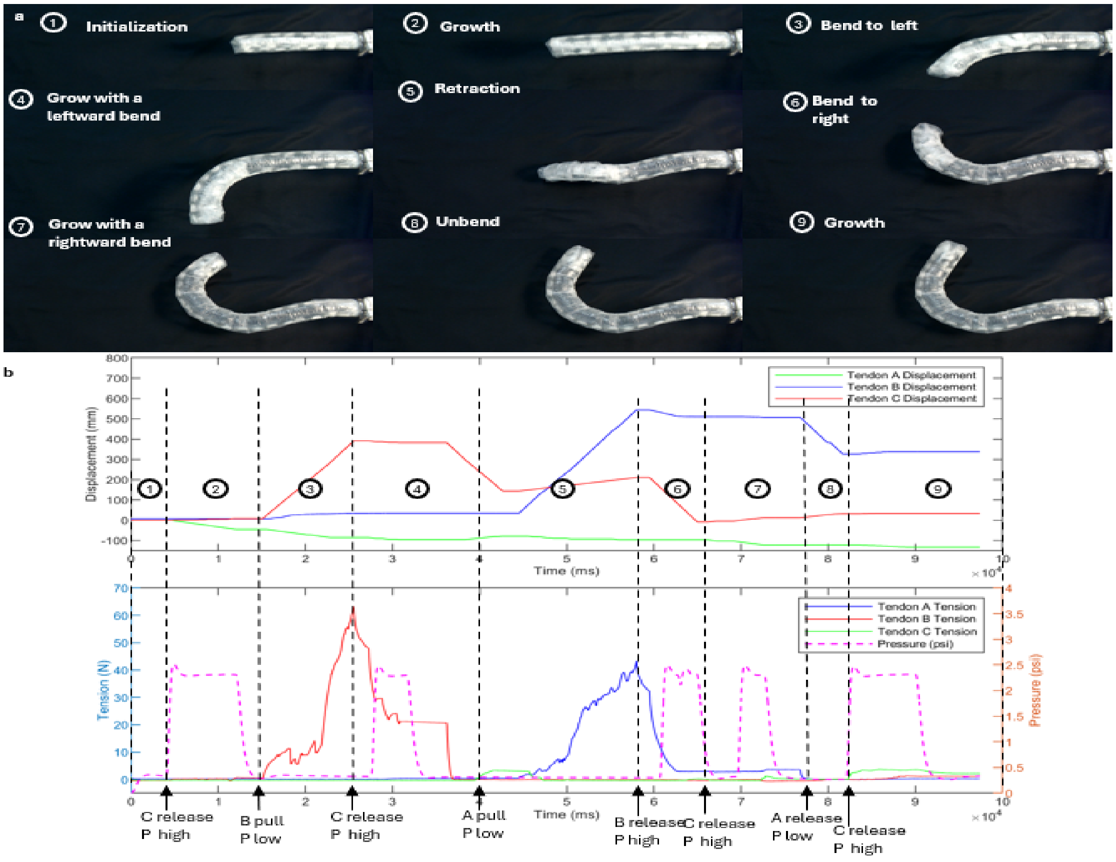

Figure S1: **Shape-morphing and Tip-growing robot a** The robot's postures at different motion stages, which was fabricated following the same procedure described in Sections 4.1 and 4.2. Each curved sheath segment has a characteristic angle of 25°, a length of 12 mm, and a gap of 8 mm between adjacent segments. **b** Control inputs for each motion, where the upper and lower tendons are denoted as A and B, respectively, and the central tendon as C.And Real-time tendon tension and pressure monitoring ① Default state: all tendons relaxed. ② Growth: tendon C is relaxed, high air pressure (2 psi) is applied, and tendons A and B relax to prevent obstruction. ③ Single-sided bending: pulling tendon B with reduced air pressure induces bending. ④ Bending-induced growth: after bending, relaxing tendon C and increasing air pressure achieve bending-induced growth. ⑤ Retraction: with pre-formed bending on tendon B, pulling tendon A closes the opposite gap, retracting the robot. ⑥ Reverse bending: relaxing tendon B and increasing pressure opens wrinkles, causing opposite bending. ⑦ Bending-induced growth: similar to step 4, bending-induced growth is achieved. ⑧ Unbending: releasing tendon A allows the robot to unbend. ⑨ Growth resumption: the robot resumes growth, continuing from its previous posture.

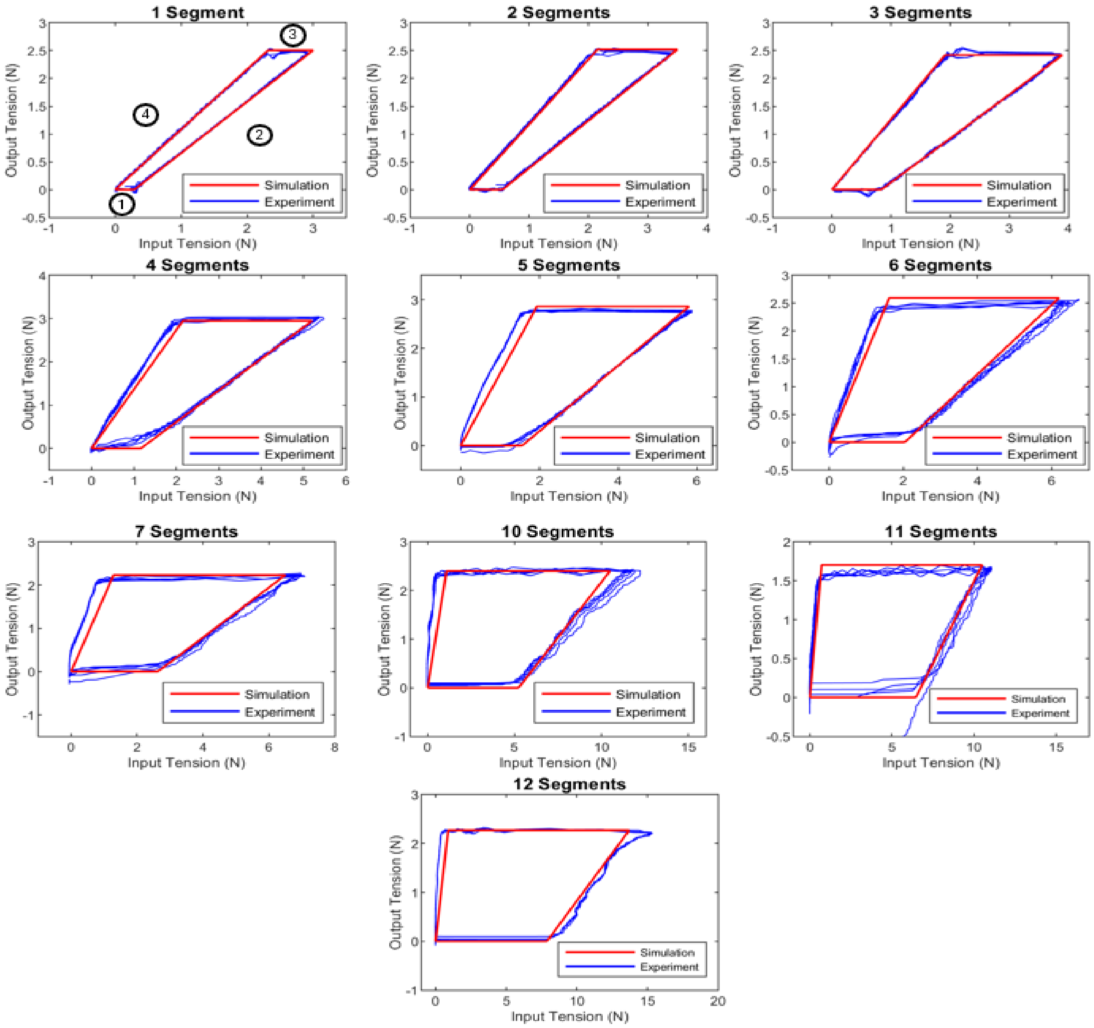

Figure S2: **Comparison between the predicted model and the experimental results.** Friction attenuation tests were conducted for a characteristic angle of $\theta = 25°$ with varying numbers of segments. The experimental setup follows the same configuration as shown in Fig. 3C. Specifically, phases ② and ④ follow the Capstan equation, while phases ① and ③ exhibit transitional friction.The transitional friction was extracted and fitted to its relationship with the total friction angle to propose an improved model. A comparison between the model and experimental results for various cases demonstrates the model's good accuracy

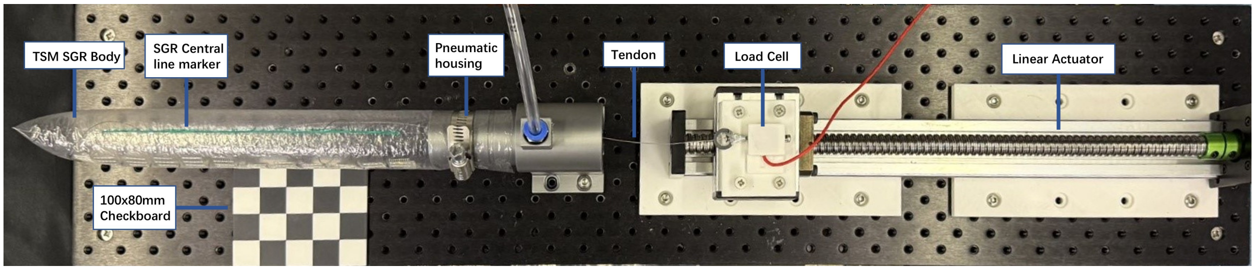

Figure S3: **Hysteresis-Assisted Shape Morphing Kinematics testbed** The experimental setup follows the procedure described in Section 4.5. A robot with 11 segments is mounted on a fixed base, and its tendons are connected to a linear actuator equipped with a load cell. A camera mounted above the setup records the shape changes introduced by each segment's bending, which are used for subsequent kinematic analysis.

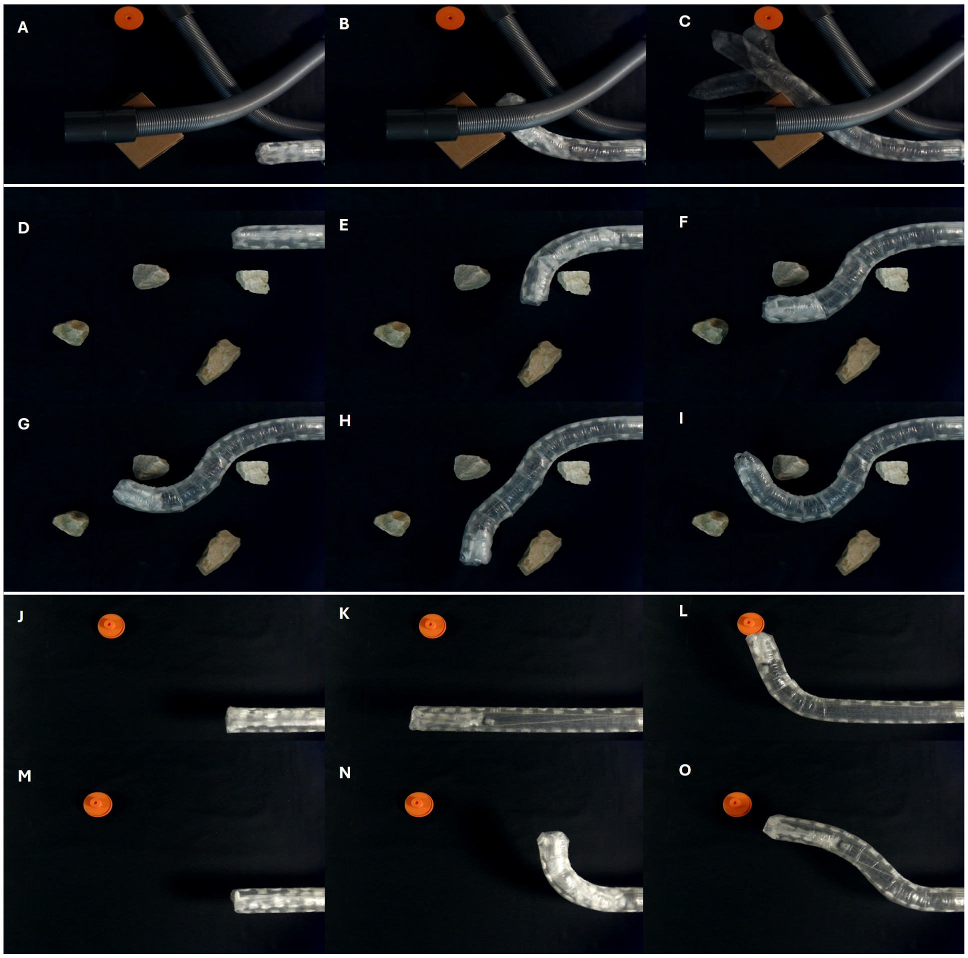

Figure S4: **Robot exploration and adaptability in real-world scenarios.** (**A**) the robot starts exploring a cluttered environment, (**B**) reaches an object by growing into a curved shape with obstacle avoidance, (**C**) explores hidden areas behind the obstacles by steering the tip after reaching the target area. (**D-I**) The robot navigates in an environment with multiple obstacles via tip growth and reversible shape morphing. (**E**) grows into a curved shape to enter the zone, (**F**) grows into an "S" shape via follow-the-leader motion to enter the free space while keeping the formed shape, (**G**) The robot grows to one direction, and (**H**) steers to the opposite direction to explore an alternative path, demonstrating reversible steering and growth. (**I**) The robot returns to the initial path. These processes can be found in Supplementary Video 10. (**J-L**) The robot approaches a target directly from below. (**M-O**) The robot first moves to the right of the target and then approaches it. This ability to achieve different postures to reach the same target allows the robot to avoid obstacles in real-world environments, reducing the reliance on precise path planning. The test process shown in the (**J-O**) can be found in Supplementary Video 11.

S1:Comprehensive Description of the Driving Process

The robot's movements are primarily driven by three tendons (A, B, and C) and pneumatic pressure control, as shown in Fig. S1. These inputs need to coordinate one another to ensure smooth operation. As illustrated in Fig. S1b, high pneumatic pressure (2 psi) is maintained during motions such as growth and unbending, while corresponding tendons are relaxed to facilitate these actions. Conversely, bending and retraction require reduced pressure to minimize resistance, with tendons tightened to provide the necessary driving forces. These principles are applied in each motion step, as detailed in Fig. S1a,b, which outlines the specific input signals and corresponding robot postures for precise and stable operations. The following section provides a step-by-step explanation of these operations to demonstrate the integration of tendon and pressure control. Straight Growth: Steps ①, ②,⑧, and ⑨. Step ① represents the default state with all tendons relaxed. In Step ②, straight growth is initiated by relaxing tendon C and applying high pneumatic pressure. Step ⑧,⑨ resumes growth from the previous posture.

External Wrinkle-induced Strreing: Step ③. Bending is induced by pulling tendon B (or A) under reduced pneumatic pressure, generating external wrinkles.

Coupled Growth and Steering: Steps ④, ⑥, and ⑦. Step ④ achieves bending-induced growth by relaxing tendSon C after bending and increasing pressure. Step ⑥ demonstrates reverse bending by relaxing tendon B while increasing pressure. Step ⑦ repeats bending-induced growth.

Tip Shortening: Step ⑤. Retraction is achieved by simultaneously pulling tendons A and B, closing gaps on both sides and reducing the robot's length.

This structured driving logic minimizes motion coupling and ensures precise, stable operations. Automatic adjustments, such as relaxing tensioned tendons when others are activated, further enhance the robot's adaptability and performance.

S2:Fitting Formula for the Transitional Phase $\mathbf{T}_p(\theta)$

For transitional friction, the critical values of the transitional phase were extracted from the experimental data (Fig. S2②). The even-numbered segments were fitted to the total friction angle using different methods, ultimately yielding an accurate cubic function as follows:

$$T_p(\theta) = 0.0006381 \times \theta^3 - 0.0025664\theta^2 + 0.1485\theta + 0.03612$$

The fitting formula was ultimately integrated into the modified Capstan model and validated against all experimental data, yielding relatively accurate predictions and confirming the model's effectiveness(see the Fig. S2 for the comparison between the simulated and experimental results.).

S3:Functional testing of the robot

The integration of tip-growth and hysteresis-assisted shape morphing enables the robot to frictionlessly navigate deep in confined spaces and dexterously avoid obstacles. The tests presented in Fig. S4 demonstrate the robot's adaptability to complex environments and its performance advantages. In Fig. S4A-C, after navigating beyond the obstacles, the robot can still be steered at the tip to explore other directions while keeping the shape of the rest of the body. Notably, in Fig. S4C, the robot not only reaches the target position but also adjusts its posture to explore the rear side of the box.

In real-world scenarios, environments often contain multiple obstacles. Fig. S4D–I showcase the robot's ability to adjust its posture and position in real-time to explore different branches in a maze-like environment. As shown in Fig. S4D–G, the robot avoids obstacles and enters a branch via tip growth in a follow-the-leader manner. Subsequently, by leveraging its tip shortening mechanism, the robot adjusts its posture and explores an alternate branch, as seen in Fig. S4H. After completing the exploration, it can even return to the original branch. The relative independence of growth and bending, combined with the unique tip shortening mechanism, allows the robot to avoid obstacles flexibly. Furthermore, these features enables the robot to adapt its posture dynamically without pre-programmed paths, making it well-suited for exploring unstructured environments.

Beyond environmental exploration, the ability to reach target objects is another critical requirement for soft growing robots. As shown in Fig. S4J–L and Fig. S4M–O, the robot's flexibility enables it to approach target objects from various postures, orientations, and angles. This capability not only helps the robot avoid obstacles to reach the target but also allows it to adjust its interaction

position and contact point with the target dynamically which can make the robot achieves an optimal force application point, improving the efficiency of its interactions. Additionally, its flexibility allows it to reach the target without relying on precise path planning.

Caption for Movie S1. Tip-to-base motion. Demonstration of sequential motion on a three-segment robot, where both bending and unbending consistently initiate from the tip and propagate toward the base.

Caption for Movie S2. Continuum robot motion demonstration. The robot performs a sequence of controlled actions including tip-initiated bending, tip-initiated unbending, and tip shortening. These motions collectively enable the formation of complex shapes, showcasing the versatility and programmability of the actuation strategy.

Caption for Movie S3. Demonstration of soft growing robot motions. This video illustrates the basic growth capability of the soft growing robot, along with two distinct bending modes—external wrinkle-induced bending and coupled growth-steering—and its tip-shortening function.

Caption for Movie S4. Continuous motion demonstration of the soft growing robot. This video demonstrates continuous shape morphing of the soft growing robot under real-time control.

Caption for Movie S5. Comparison between Inverted Zigzag TSMs and traditional TSMs. The Inverted Zigzag TSMs enable effective and predictable tip-to-base motion. In contrast, bending and contact behavior in traditional TSMs are random and unpredictable.

Caption for Movie S6. Comparison between robot motion and kinematic simulation. Real-time comparison between the actual robot motion and the corresponding kinematic simulation results, demonstrating the accuracy of the proposed kinematic model.

Caption for Movie S7. Three-segment robot and 27 configurations. Demonstration of a three-segment robot achieving 27 distinct configurations through sequential actuation. The video presents both the robot's motions alongside the corresponding real-time control inputs.

Caption for Movie S8. Demonstration of tip-shortening functionality. The robot dynamically shortens its tip in response to task requirements and environmental constraints. This capability enables the robot to avoid obstacles and reach designated targets in real time.

Caption for Movie S9. Real-time exploration of unstructured environments by the growing robot. The robot demonstrates real-time shape adaptation to bypass obstacles, combining tip growth and bending to reach designated targets. The tip-shortening capability further enables the robot to retract to previous nodes and switch to alternative paths when needed.

Caption for Movie S10. Exploration of unstructured environments by the growing robot. The robot performs real-time exploration in various unstructured environments, demonstrating multiple capabilities including obstacle avoidance, shape morphing, and retraction. These functions are achieved through real-time control strategies that enable the robot to actively adapt its body configuration during navigation.

Caption for Movie S11. Multi-path access to the same target. The robot is capable of reaching the same target object via different motion trajectories and growth paths, demonstrating flexible path planning and execution in complex environments.
